# Supplementary material for: Chromothripsis during telomere crisis is independent of NHEJ, and consistent with a replicative origin
Source: Genome Res. 2019 May;29(5):737–49. doi: 10.1101/gr.240705.118 (PMC6499312; doi:10.1101/gr.240705.118)
Supplement: Supplemental Material [file supp_gr.240705.118_Supplemental_file_1.zip › contigs/annotated_contigs/DB55/contig.2.DB55_length_469_mean_cov_4.51172707889.docx]

**DB55_length_469_mean_cov_4.51172707889**

```
GCAGTGAGTACGAAATACTATGAAAAAATAGTTTTCTCTTAATATCTCCTAGTATTCTATCAATCATGTAAGCTATCT|ACTACCAATT
                                                                               >chrX:325
CCTTATGCCATTTTCCAACTCTGATAGTATATTTTAAAAGCCAGGTTTTTGTGTGAAAAAGCAAGTTTAAAAAAGTAAAGAGGAAATAA
00956-32501280 + E=1e-177
AAGTCAGTATGGATGCCTAGGCACTTCTGTTAGCTCTTATTTCCTGCATACTTAATACGTCCAAAGTACTGTGGAGGAATACAAGAAAT

GAGATTTACTCCCTGGTGCCAGTTCGGATATAATGCATACACATGTGCAATTATTTGAATTAATTTCAAATTAACAAAATCAACTGCTA

TGTAATTTCATATGCTACATTTTTCATTCTATATATATATATATA|TA|CATTAATTCTGGAAATGATATTATATGGTGAATTCTTTAA
                                              >chrX:32502043-32502112 + E=6e-30
ATATTTACAAAGAAAAGCAATAGGATA
```
